# Micronutrient Intakes of British Adults Across Mid-Life: A Secondary Analysis of the UK National Diet and Nutrition Survey

**DOI:** 10.3389/fnut.2018.00055

**Published:** 2018-07-19

**Authors:** Emma Derbyshire

**Affiliations:** Nutritional Insight Limited, London, United Kingdom

**Keywords:** micronutrients, females, young adults, selenium, iron, potassium

## Abstract

**Background:** There is a tendency to report micronutrients intakes collectively for adults, with broad age ranges being used. This means that certain sub-population groups such as young adults are often overlooked. The objective of the present article was to derive and evaluate micronutrient intakes across UK adults in their twenties, thirties, forties and fifties.

**Methods:** A secondary analysis of the UK National Diet and Nutrition Survey (years 1–6) was undertaken. Data from *n* = 3,238 adults was analyzed and micronutrient intakes from food sources (excluding supplements) derived as a percentage of the Reference Nutrient Intake (RNI) and percentage below the Lower Reference Nutrient Intake (LRNI) for males and females aged 20–29, 30–39, 40–49, and 50–59 years. Mean intakes were used in instances where this data was unavailable (for vitamins D and E).

**Results:** Sizeable gaps were found for magnesium with 19% of young people in their twenties having intakes below the LRNI. Amongst UK females intakes of 9 micronutrients (riboflavin, vitamin B6, B12, folic acid, calcium, iron, magnesium, potassium, and iodine) were significantly lower than males aged 20–59 years (*p* < 0.001) expressed as a percentage of the RNI. Young adults in their twenties had significantly lower (*p* < 0.05) intakes of 8 micronutrients (vitamin A, riboflavin, folic acid, calcium, magnesium, potassium, iodine, and copper) expressed as a percentage of the RNI compared with adults in their thirties, forties and fifties. There were also considerable gaps in dietary selenium intakes with 50.3% females and 25.8% males having total intakes beneath the LRNI. A quarter of women had iron (25.3%) and potassium (24.3%) intakes below the LRNI.

**Conclusions:** UK females and younger adults appear to be particularly vulnerable to micronutrient shortfalls from food sources alone. Clearly, improvements in dietary quality are needed across mid-life. Alongside this, fortification and supplementation strategies may be considered to help adults achieve dietary targets at this life-stage when they should be at their nutritional prime.

## Introduction

Micronutrient insufficiencies are not a thing of the past. In the twenty-first century these continue to exist with around two billion people worldwide having micronutrient inadequacies (1). This includes those living in the developed world in regions such as the United Kingdom, United States and Germany where nutrient-poor food is abundant and consumed on a regular basis, ultimately impacting on healthcare costs (1, 2). Micronutrients are substances such as vitamins or minerals that are required by the human body in miniscule amounts, described by the World Health Organization as “magic wands” that help the body to produce enzymes and hormones needed for growth and development (3). Healthy eating habits providing key dietary components alongside physical activity across mid-life can help to maintain health and reduce or prevent age-related chronic diseases (4).

In general dietary intake surveys and studies tend to focus on the micronutrient profiles of adults collectively. The UK National Diet and Nutrition Survey Rolling Programme (NDNS-RP) provides mean daily intakes for vitamins and minerals for adults aged 19–64 years (5). In the U.S. the National Health and Nutrition Examination Survey (NHANES) reports micronutrient intakes for those aged ≥20 years (6). Some other work has looked at dietary patterns in baby boomers (adults aged ages 46–64 years) but did not investigate the habits of younger adults (7). The Oxford Dictionary defines mid-life as the “period of age between young adulthood but before the onset of old age” typically 45–65 years but overlooks early adulthood i.e., those in their twenties and thirties (8). So, methodologically there appears to be inconsistencies in terms and the age ranges used to define what constitutes this life stage with younger adults tending to be overlooked.

This is concerning given the significance of the middle-years of life. Micronutrient intakes in early adulthood are particularly important as these are typically the years of conception and childbearing (9). An adequate micronutrient profile is not only important for fertility but also to prepare the body for the extensive physiological demands should pregnancy occur (9). Nutritional intakes in mid-life can help to future-proof health against debilitating and chronic diseases that can occur in later life (4). For example, research suggests that physiologic aspects of age-related cognitive decline can begin as young as 18 years with healthy educated adults in their twenties and thirties also showing signs of deterioration (10). Yet current nutritional science appears to overlook the role of micronutrients in mid-life. There is clearly a lack of research documenting how habitual micronutrient profiles change across the decades of mid-life.

The present article undertakes a secondary analysis of the UK NDNS-RP breaking down and evaluating daily micronutrient profiles of UK women and men across their twenties, thirties, forties, and fifties. This novel approach will add to the evidence-base providing new insights on micronutrient profiles across mid-life.

## Methods

This is a secondary analysis of the UK NDNS-RP using data from years 1 to 4 (2008/9 to 2011/12) and 5 to 6 (2012/13 to 2013/14). The UK survey collects data throughout the year across the UK (England, Northern Ireland, Wales and Scotland). In this survey selected participants were asked to keep an A5 diary record of everything that they ate and drank in and outside the home over 4 consecutive days with the start date being randomly allocated so weekends were included. Diaries provided photographs of 15 frequently consumed foods as small, medium and large portion sizes. Recorded food intakes were entered into a dietary assessment system known as DINO (Diet In Nutrients Out). The food composition data used was Public Health England's NDNS Nutrient Databank which was incorporated into the DINO system. The Food Standards Agency Food Portion Sizes book provided weights for unprocessed foods whilst weights for manufactured products were extracted from retailer websites. Using this data average micronutrient intakes were estimated from the data that was collected. Additional methodological details can be found in section 7 of the full report (11) and Appendix A of the supplementary NDNS information (12).

Dietary intakes of vitamins and minerals were investigated across mid-life. Age ranges were divided into: 20–29, 30–39, 40–49, and 50–59 years. Mean intakes were calculated from diary information related to micronutrients from food sources only (intake from supplements was excluded). Mean intakes were calculated where reference nutrient intake (RNI) and Lower Reference Nutrient Intake (LRNI) data was not available (for vitamin D and E). The RNI is the amount of a nutrient that is enough to ensure that the needs of nearly all the group (97.5%) are being met. By definition, many within the group will need less (13). Particular focus was also given to the percentage of individuals with micronutrient intakes below the LRNI, as this is the level below which deficiencies are most likely to occur (13). Comparisons across gender and mid-life age groups were made.

### Statistical analyses

Statistical analysis was performed using R studio v 3.4. Data from years 1 to 6 was combined. New weights were computed to account for higher samples size in years 1–4. Data was filtered to include the subpopulation of interest (adults 20–60 years old). Outcomes were categorized as being binomial or continuous. Binomial data was displayed as proportions and standard errors. Continuous data was presented as means and standard errors (SE). Confidence intervals (95% CI) were also calculated.

Pearson's Chi square (Rao and Scott adjustment) was used to assess associations between gender and mean daily intake below the LRNI and percentage of RNI achieved. For continuous data (vitamin D and E intakes) *t*-tests were used. Geometric means were applied as results showed that variables were not normally distributed. Log transformation was performed to reduce bias due to the skewed nature of the data. For all statistical analyses, a p value less than 0.05 was used to denote statistical significance.

Generalized linear regression was used to assess associations between age and micronutrient intakes. Individuals in their twenties were used as the reference category. All these tests were performed using the survey package in R studio account for the sampling design of the NDNS-RP survey.

## Results

### Participants

The NDNS-RP data for years 1 through to 4 contains diary records and mean daily intakes for *n* = 6,828 individuals. The survey for years 5 and 6 provides records for *n* = 2,546 individuals. After combining data sets and filtering the data to include the subpopulation of interest (adults in mid-life aged 20–59 years) the final data set contained records for *n* = 3,238 individuals (*n* = 2,377 in years 1 to 4 and *n* = 861 in years 5 and 6). As shown in Table 1, after adjustments (using weight) each of the four age groups were approximately equal. The number of individuals who used supplements during the 4-day diary use was *n* = 708 (22%). Supplement data was excluded and food data only analyzed from all 3,238 participants.

**Table 1:** Percentage of participants in each age group.

**Age groups**	**% per age group (SE)**		**% of age group per sub-population**
	**Males**	**Females**	
20–29 years	50.6 (2.6)	49.4 (2.6)	25.8
30–39 years	49.8 (2.12)	50.2 (2.12)	24.3
40–49 years	49.4 (2.03)	50.6 (2.03)	27.1
50–59 years	49.4 (2.38)	50.6 (2.38)	22.8
Overall	49.8 (1.19)	50.2 (1.19)	100

### Findings by gender

**Figure 1:**
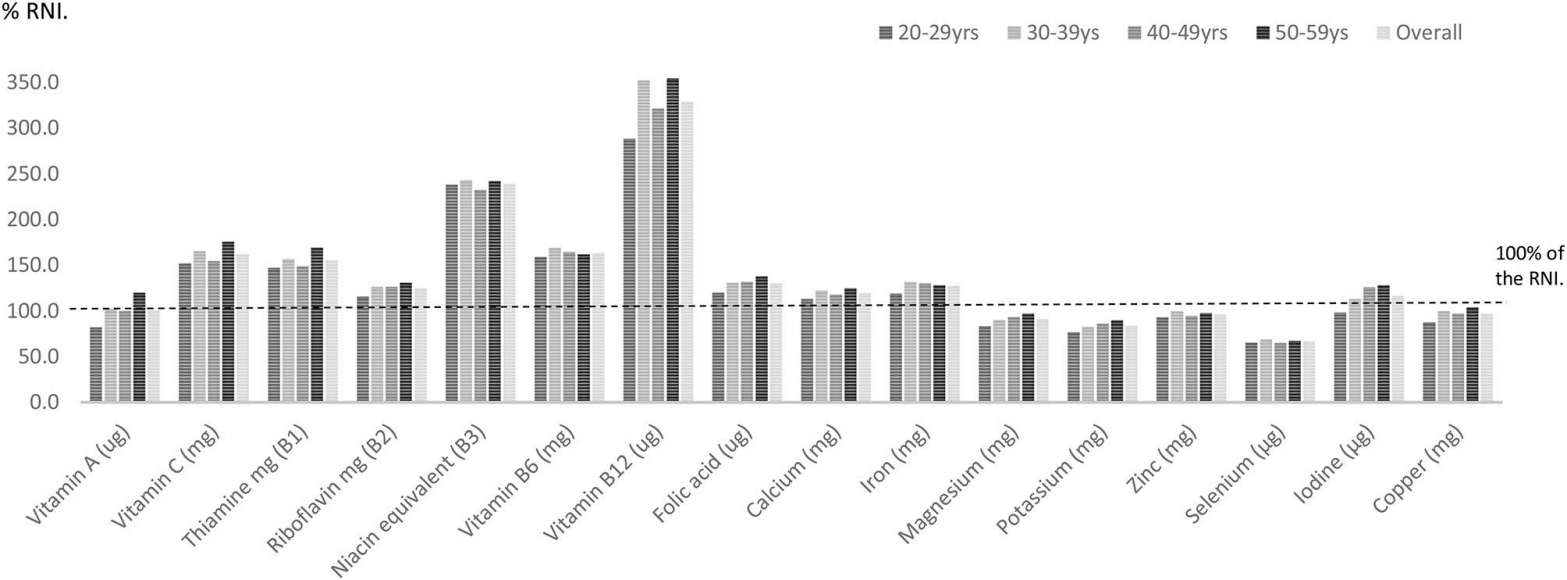
Percentage RNI for UK males across mid-life.

Females in their mid-life years were more likely to have micronutrient shortfalls than males. Amongst males some shortfalls were evident. Vitamin A intakes expressed as a percentage of the RNI were statistically significantly lower amongst males aged 20–59 years compared with females in this age category (*p* = 0.002). Zinc intakes were also significantly lower in UK males compared to women (96.1% RNI vs. 101% RNI) (*p* < 0.001). Average intakes of five micronutrients—magnesium, potassium, zinc, selenium, and copper expressed as a percentage of the RNI also fell below dietary targets for males aged 20–59 years (Figure 1; Table 2).

**Table 2:** Percentage RNI for vitamin and mineral intakes by gender and age.

	**Gender % RNI**			**Age group % RNI**				RNI^**^	
	**Males**	**Females**	***P***	**20+**	**30+**	**40+**	**50+**	**Males**	**Females**
**VITAMINS**									
Vitamin A (μg) (retinol equivalents)	99.5	115	0.002^*^	91.9	107^†^	108^†^	126^†^	700	600
Vitamin C (mg)	161	158	0.22	148	159	155	181^†^	40	40
Thiamine mg (B1)	155	150	0.05	145	155^†^	150	162^†^	1.0	0.8
Riboflavin mg (B2)	125	116	<0.001^*^	112	120^†^	122^†^	128^†^	1.3	1.1
Niacin equivalent (B3)	239	229	0.02^*^	231	235	228^†^	245	16.5	13.2
Vitamin B6 (mg)	163	138	<0.001^*^	148	152	150	152	1.4	1.2
Vitamin B12 (μg)	327	255	<0.001^*^	265	290^†^	155^†^	181^†^	1.5	1.5
Folate (μg)	130	104	<0.001^*^	108	115^†^	117^†^	125^†^	200	200
**MINERALS**									
Calcium (mg)	119	97.1	<0.001^*^	103	108	107	104^†^	700	700
Iron (mg)	127	67.8	<0.001^*^	84.0	90.6^†^	87.8	113^†^	8.7	14.8
Magnesium (mg)	90.8	80.2	<0.001^*^	79.2	85.1^†^	86.4^†^	91.7^†^	300	270
Potassium (mg)	93.5	68.6	<0.001^*^	69.9	74.6	76.9	82.5^†^	3,500	3,500
Zinc (mg)	96.1	101	<0.001^*^	94.6	101^†^	97.4	103^†^	9.5	7.0
Selenium (μg)	66.6	66.0	0.933	65.6	67.3	64.4	68.2	75	60
Iodine (μg)	115	88.7	<0.001^*^	88.5	99.2^†^	106^†^	114^†^	140	140
Copper (mg)	96.9	80.0	<0.001^*^	82.8	88.9^†^	87.5^†^	93.9^†^	–	–

* *p < 0.05*.

** *PHE (14) and DH (13)*.

† *Statistically significant differences compared with adults in their 20 s*.

*Data presented as geometric means*.

**Figure 2:**
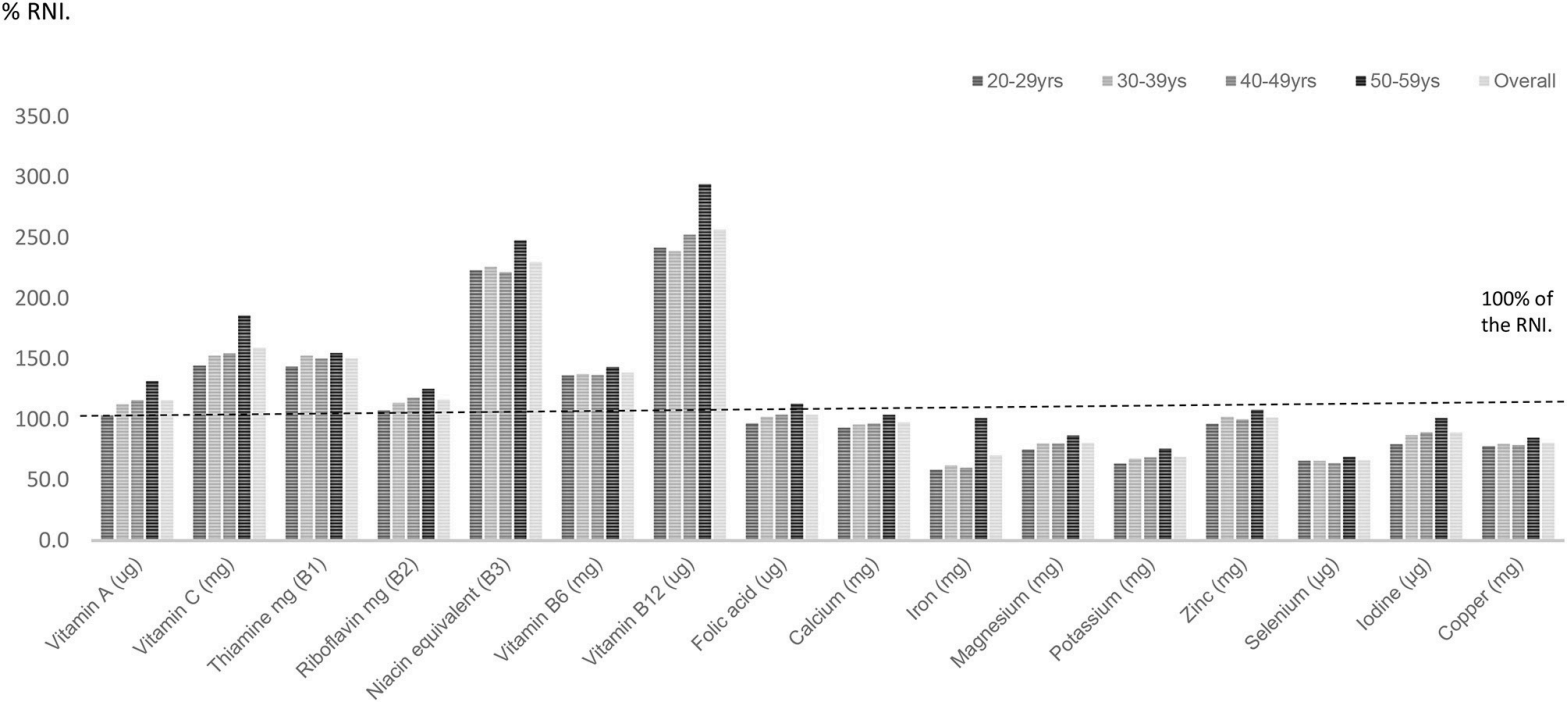
Percentage RNI for UK females across mid-life.

For UK females, riboflavin, vitamin B6, B12 and folic acid intakes were statistically significantly lower amongst UK females aged 20–59 years compared with males of this age (*p* < 0.001) (Table 2). For the minerals, calcium, iron, magnesium, potassium, and iodine intakes expressed as a percentage of the RNI were significantly lower amongst women aged 20–59 years compared with males of this age (*p* < 0.001). Average intakes of seven micronutrients—calcium, iron, magnesium, potassium, selenium, iodine, and copper intakes as a percentage of the RNI fell below dietary benchmarks (Figure 2; Table 2).

A substantial proportion of UK females and males aged 20–59 years (50.3 and 25.8%, respectively) had selenium intakes below the LRNI. A quarter (25.3%) of women had iron intakes below the LRNI and over one in 10 adults (15% males and 12% females) had magnesium intakes below the LRNI. A quarter (24.3%) of women and 10% of males had potassium intakes below the LRNI. Magnesium shortfalls were significantly higher (*p* < 0.05) amongst younger adults in their twenties compared with older adults with around 1 in 5 (19%) having magnesium intakes below the LRNI (Table 3). More specifically, amongst females one in 10 had riboflavin, magnesium and iodine (12.6, 11.5, and 11.3%, respectively) below the LRNI.

**Table 3:** Percentage of adults below the LRNI for vitamin and mineral intakes by gender and age.

	**Gender % below LRNI**			**Age group % below LRNI**					LRNI^**^	
	**Males**	**Females**	***P***	**20+**	**30+**	**40+**	**50+**	***P***	**Males**	**Females**
**VITAMINS**										
Vitamin A (μg) (retinol equivalents)	11.3	6.75	<0.001^*^	13.3	7.06^†^	9.41	5.62^†^	<0.05^*^	300	250
Vitamin C (mg)	0.98	1.33	–	0.65	1.87	1.12	1.01	–	10	10
Riboflavin mg (B2)	3.87	12.6	<0.001^*^	10.1	8.72	7.24	6.82	>0.05	0.8	0.8
Vitamin B12 (μg)	1.15	2.14	–	2.69	1.27	1.06	1.58	–	1.0	1.0
Folate (μg)	1.77	4.36	0.0013^*^	3.25	3.79	3.06	2.10	>0.05	100	100
**MINERALS**										
Calcium (mg)	4.90	8.80	0.0002	9.36	5.95	6.76	5.15^†^	<0.05	400	400
Iron (mg)	1.10	25.3	<0.001	16.6	14.1	17.3	5.06^†^	<0.05	8.0	4.7
Magnesium (mg)	14.2	11.5	0.097	18.5	10.9^†^	12.3^†^	9.7^†^	<0.05	190	150
Potassium (mg)	10.0	24.3	<0.001	24.7	15.6^†^	17.8^†^	9.79^†^	<0.05	2,000	2,000
Zinc (mg)	8.20	5.20	<0.001	8.58	5.98	6.33	5.85	>0.05	5.5	4.0
Selenium (μg)	25.8	50.3	<0.001	39.0	36.5	41.0	35.0	>0.05	40	40
Iodine (μg)	5.70	11.3	<0.001	14.7	7.37	6.16^†^	5.59^†^	<0.05	70	70

* p < 0.05

** *PHE (14) and DH (13)*.

† *Statistically significant differences compared (with/to) adults in their 20 s*.

–*Data sample size too small to determine significance*.

As shown in Table 2 men aged 20–59 years were significantly more likely to have vitamin A intakes below the LRNI compared with women (11.0 vs. 6.7%, respectively, *p* < 0.001). A significantly higher proportion of females, however, had folic acid intakes below the LRNI (4.36 vs. 1.8%, respectively, *p* = 0.0013). Mean daily intakes of vitamin D were significantly higher amongst UK males compared to females (2.4 vs. 1.9 μg; *p* < 0.001). Mean daily vitamin E intakes were also significantly higher amongst UK males aged 20–59 years (9.3 mg) compared to intakes of 7.8 mg in UK females of a similar age (*p* < 0.001).

### Findings by age

Adults in their twenties had significantly lower vitamin A, riboflavin and folic acid intakes (*p* < 0.05), expressed as a percentage of the RNI when compared to adults in their thirties, forties and fifties. Intriguingly, vitamin B12 intakes (also as a percentage of the RNI) were statistically significantly lower amongst adults in their forties and fifties compared to those in their twenties (*p* < 0.05; Table 2). For the minerals, young adults aged 20–29 years had significantly lower calcium, magnesium, potassium, iodine, and copper intakes (expressed as a percentage of the RNI) compared with adults in their thirties, forties and fifties (*p* < 0.05; Table 2). For iron, adults in their twenties had significantly lower intakes compared to those in their thirties and fifties but no differences were observed when compared against adults in their forties.

**Figure 3:**
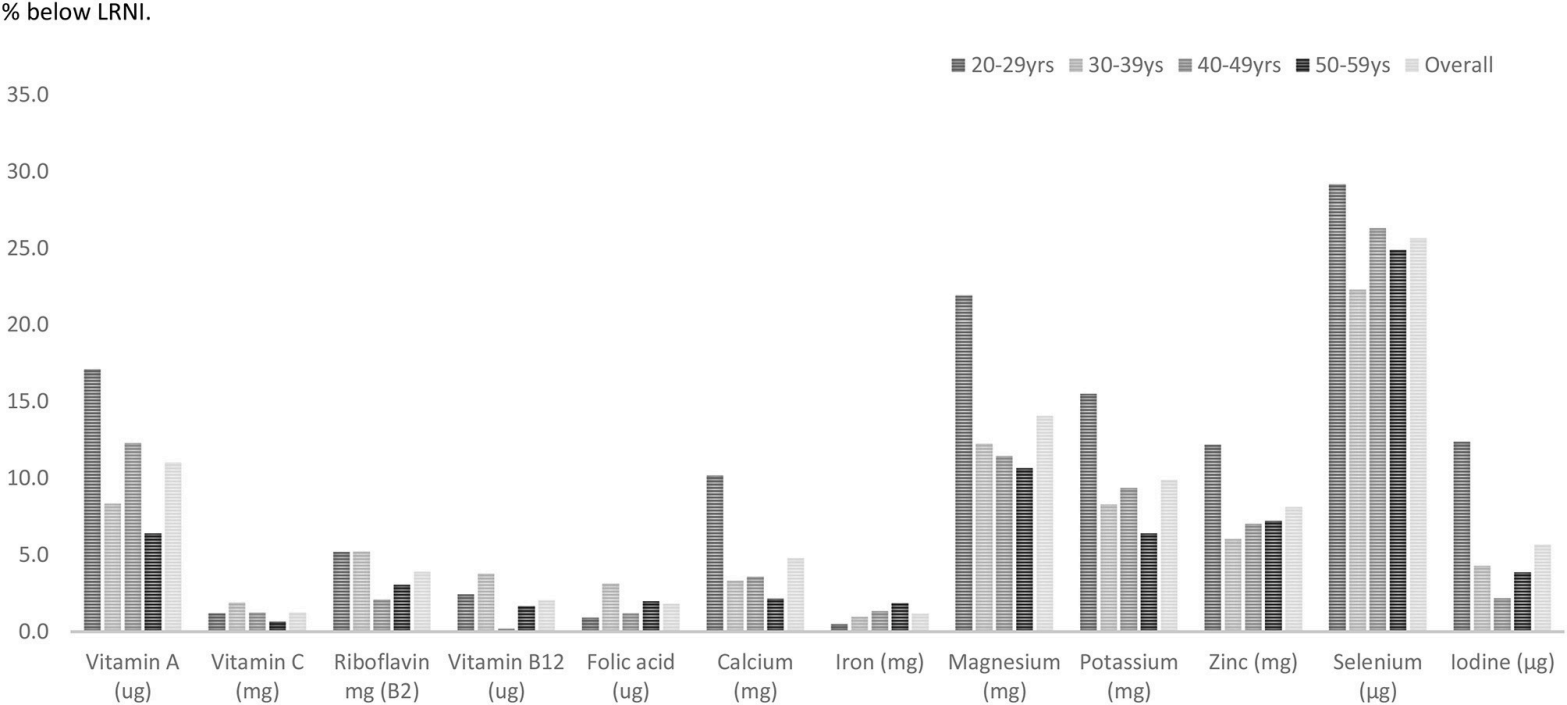
Percentage of males with vitamin and mineral intakes below the LRNI across mid-life.

Amongst males just under one-third (29%) aged 20–29 years had selenium intakes below the LRNI (Figure 3). One in five (21.9%) males in this age category had magnesium intakes below the LRNI and 17% had vitamin A intakes below the lower reference nutrient standard. Statistical analysis showed that a significantly higher proportion of males in their twenties had vitamin A intakes below the LRNI compared to those in their thirties and fifties (*p* < 0.05). Magnesium and potassium intakes were also more likely to fall below the RNI amongst males in their twenties (*p* < 0.05). Fifteen percent of men in their twenties had iodine intakes below the LRNI; significantly more than those in their forties and fifties.

**Figure 4:**
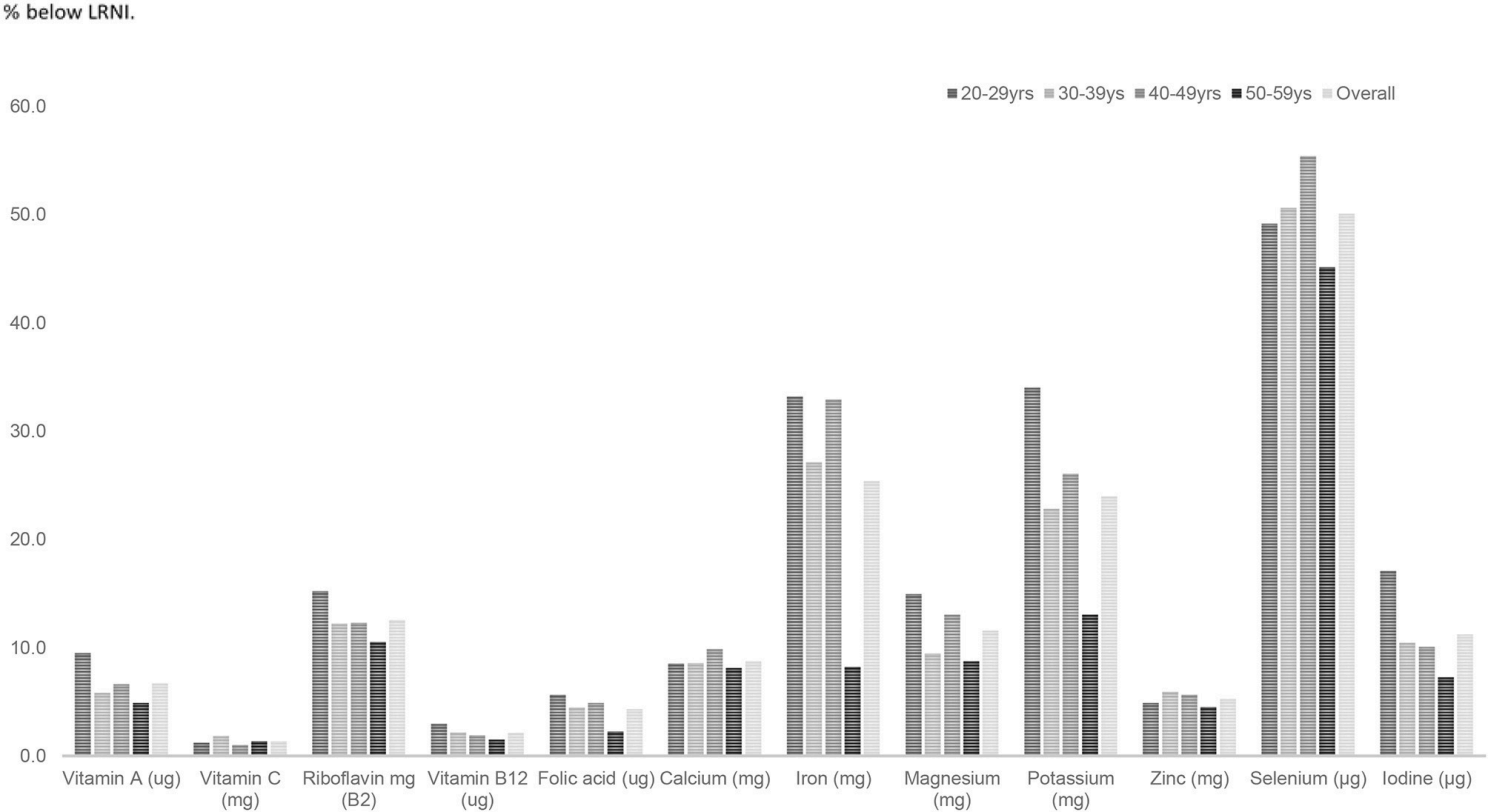
Percentage of females with vitamin and mineral intakes below the LRNI across mid-life.

Amongst females fifty per cent of females aged 20–59 years had selenium intakes below the LRNI and a quarter (25.3%) in this age group had inadequate iron intakes. Amongst females aged 20–29 years 34% had potassium and 17% had iodine intakes below the LRNI (Figure 4). A significantly higher proportion of females aged 20–29 years had magnesium, potassium and iodine intakes below the LRNI (*p* < 0.05) compared to those in their thirties, forties and fifties. Significantly more (9.4%) women aged 20–29 years had calcium intakes below the LRNI compared to 5.2% of women in their fifties (*p* < 0.05). More women in their twenties (16.6%) also had iron intakes below the LRNI compared with those in their fifties (5.5%; *p* < 0.05). With regard to vitamin D, mean intakes (1.9 μg daily) were significantly lower amongst UK adults in their twenties compared to those in their forties and fifties (mean daily intake; 2.4 μg) (*p* < 0.05).

## Discussion

Taken together, this secondary analysis highlights a number of important findings. The article sought to evaluate micronutrient intakes across mid-life and according to gender and a number of distinct differences are apparent. Firstly, there appears to be clear differences in the micronutrient profiles of UK males and females aged 20–59 years. Sizeable gaps were found for magnesium intakes compared with intake targets, particularly amongst young adults in their twenties. Research to date suggests that magnesium deficiencies could be a significant contributor to low-grade inflammation which typically underpins conditions such as diabetes, cardiovascular disease and hypertension (15). At a cellular level magnesium is thought to have a valuable role in the regulation of telomere function, integrity, and structure with telomere dysregulation being linked to aging and age-related disease such as cancer (16).

Amongst UK males, vitamin A and zinc shortfalls were apparent. The clinical importance of vitamin A is becoming increasingly clear with evidence of its role emerging in immune competence, tissue differentiation and the visual cycle (17). The trend toward males having lower vitamin A intakes may have been driven by several different factors. Data from Years 5 and 6 of the UK NDNS shows that men's mean intakes of fruit and vegetables were slightly lower at 3.9 portions daily compared with 4.1 portions daily amongst women aged 19–64 years (5). It has also been found that fruit and vegetable variety tends to be lower in men, especially in instances where education and social class is lower (18). Turning to zinc, this micronutrient is a known antioxidant with research showing that fertile males tend to have higher seminal zinc levels than their infertile counterparts (19). Zinc also has important catalytic, structural, and regulatory roles helping to support immunity and avert age-related diseases (20). Zinc shortfalls are somewhat surprising to see, especially amongst males given that meat and meat products are one of the main providers of zinc (21). It is possible that younger men in their twenties are eating less meat which could have contributed to lower zinc intakes in this age group. This is an important finding and worthy of consideration in the context of public health given current trends toward plant-based diets (22).

Amid women, a number of micronutrient shortfalls were also evident. There are a number of possible underpinning reasons behind these findings. A recent survey of 1,035 social media tweets typically used by young adults found that 67.2% related to body image, eating disorders, fitness, food or dieting (23). This, in turn, could have wider ramifications impacting on dietary habits and micronutrient profiles of young women. Emerging food trends and the avoidance of food groups could be impacting on micronutrient intakes (24). For example, the consumption of eggs, milk, and dairy correlates strongly against female nutritional iodine status (25). Veganism has also been found to impact on vitamin D, calcium, and vitamin B12, iodine and selenium intakes (26, 27). UK females having diets lower in red meat (<40 g daily) have reduced micronutrient intakes, especially zinc and vitamin D (28). Alongside this, factors such as the level of perceived control over life may be important predictors of dietary quality, especially amongst women of lower educational attainment (29). Meal frequency has also been positively associated with diet quality and micronutrient intakes (30), indicating that skipping meals or snacking rather than eating main meals may also impact on micronutrient intakes.

In relation to age, findings showed that younger adults appeared most susceptible to micronutrient shortfalls. Similar findings have been observed in other publications. For example, an earlier report concluded that this could be attributed to people in their fifties having more time to cook and prepare food from scratch (31). Forty per cent of adults in their twenties had selenium intakes below the LRNI, a third of young women aged 20–29 years (33%) had iron intakes below the LRNI and a quarter (24.7%) had potassium intakes below lower recommended nutrient thresholds. Overall, more than 1 in 10 young adults (aged 20–29 years) had four micronutrients below the LRNI (vitamin A, iron, magnesium, and iron). These findings are concerning. For example, selenium is an essential micronutrient associated with human health outcomes such as cancers, cardiovascular, and autoimmune disease (32, 33). Amongst UK women low whole-blood selenium levels have recently been linked to increased pre-eclampsia and pregnancy-induced hypertension risk (34) whilst iron deficiency has been associated with higher rates of depression in pregnancy (35). Regarding potassium low fruit and vegetable intakes may be one factor contributing to declining intake. Potatoes alongside white vegetables are important dietary contributors of potassium although the additional use of salt should be discouraged (36).

Clearly, improving diet quality through mid-life may help to protect health, prevent chronic disease, and disability and enhance economic productivity (1). In other regions, scientists have looked into muli-vitamins and minerals as a means of improving micronutrient profiles. For example, research conducted with young Australian adults (18–40 years) found that multi-vitamin and mineral supplementation significantly improved blood B-vitamin levels whilst lowering homocysteine levels (*p* < 0.01) and after 4 weeks notable improvements in mood (*p* = 0.018) were observed (37). In the United States NHANES analysis showed that in large populations where micronutrient sufficiency is not being achieved from food sources multivitamin/mineral supplements can serve as a practical means to improve micronutrient status (51% adults took multivitamin/mineral supplements containing ≥9 micronutrients) without increasing intakes beyond upper limits (38). Selenium supplementation in some geographical areas with soil selenium deficiency is also now recommended as part of public health policy (39).

## Limitations and future directions

Whilst the present publication identified micronutrient profiles of UK adults across mid-life, similar data to compare findings against is lacking. This emphasizes the need for harmonized definitions and data collection methods both across European nations and further afield in order to delve and better understand nutritional needs across mid-life in the twenty-first century. Given growing market trends there is also a need to better consider and analyze the impact of changing food trends on micronutrient profiles (40). As with most dietary assessment tools it should be recognized that factors such as under-reporting can occur, especially amongst individuals with a higher body weight (BMI > 25 kg/m^2^) (41).

Subsequently, future research should also investigate micronutrient status using blood biomarkers. The UK NDNS only collects blood from a sub-sample of the population and when divided over the mid-life decades would not generate enough statistical power to warrant analysis in the present publication. There is certainly potential to “focus in” on particularly vulnerable sub-samples such as young adults and females during early adulthood and assess micronutrient blood biomarkers in these population groups. For example, one Australia study, comprised of 308 females aged 18–35 years, found that iron deficiency anemia, unspecified anemia, hypoferritinemia, and low vitamin B12 concentrations (<120 pmol/L) was present in 3, 7, 33.9, and 11.3% of participants, respectively (24). In the same study serum copper and selenium concentrations were below reference ranges in 23 and 11% of females, correspondingly (24).

The current research found that females and young adults are at particular risk of micronutrient shortfalls. In an obesogenic environment, where the public are being encouraged to reduce their energy intakes, it is important to ensure that the micronutrient profile of diets is sustained (42). The same concept applies to specific food groups, for instance, in cases where red meat reduction is being advocated (28). It has been predicted that over the next few years micronutrients of concern are likely to remain similar although ongoing dietary trends could further impact on iron and calcium intakes (42). As concluded by the World Health Organization, as tiny as the amounts are, the consequences of micronutrient shortfalls can be severe with vitamin A, iron and iodine being of particular significant importance in terms of global public health (43). It is imperative to continue raising awareness about the important of healthy and balanced diets providing an adequate micronutrient density. The implications of “cutting back or out” certain food groups also needs to be communicated, especially to younger generations who are now being strongly influenced by social media that is not subject to peer review or monitoring systems. Alongside this, the role of multivitamin and mineral supplements and value of daily compliance also warrants practical consideration. For example, in the UK there is a Government health message to supplement with 10 μg vitamin D between Autumn and Spring to protect bone and muscle health (44).

Finally, in terms of extended roles, the impact of micronutrients in relation to gut microbiota is now beginning to emerge. For example, researchers have found that acute vitamin A deficiency can impact on bacterial community structure with *Bacteroides vulgatus* increasing in abundance in the absence of vitamin A (45). Clearly, further research is needed on individuals with suboptimal rather than acute nutritional shortfalls to see if such inter-associations still exist and in relation to the potential role(s) of other micronutrients. Other recent work has found that nutrients such as iron, zinc, and magnesium are positively associated with sleep duration with potential mechanisms appearing to involve the effects that micronutrients have on neurotransmitters and the expression of circadian genes (46). Ongoing work is undoubtedly needed to monitor the ramifications of ongoing food trends, food fortification, and updated public policies.

## Conclusions

The present review has identified that micronutrient shortfalls are evident across mid-life in UK adults. These shortfalls are more prominent amongst females and young adults in their twenties. This is of particular concern given that early adulthood is a time to be in the “nutritional prime” of life preparing for parenthood. Mid-life is also a time to lay the foundations of good health in preparation for later life. Given that current gaps exist between micronutrient intakes and requirements the importance of a healthy and balanced diet needs further reiteration. This particularly applies in relation to the elimination or substitution of key food groups. Alongside this, the value of multivitamin and mineral supplements and food fortification strategies should not be overlooked in the context of today's modern lifestyles.

## Author contributions

ED is responsible for the planning, analysis, and the write-up of the publication. ED critically reviewed the manuscript and approved the final version submitted for publication.

### Conflict of interest statement

ED received funding from the Health & Food Supplements Information Service (HSIS). The article was written by the author alone and HSIS had no role in writing the publication.

## References

[1] Peter S Eggersdorfer M van Asselt D Buskens E Detzel P Freijer K. Selected nutrients and their implications for health and disease across the lifespan: a roadmap. Nutrients (2014) 6:6076–94. 10.3390/nu6126076 25533014 PMC4277016

[2] Hoeft B Weber P Eggersdorfer M. Micronutrients - a global perspective on intake, health benefits and economics. Int J Vitam Nutr Res. (2012) 82:316–20. 10.1024/0300-9831/a000125 23798049

[3] WHO Micronutrients. Available online at: http://www.who.int/nutrition/topics/micronutrients/en/ (2018).

[4] Chedraui P Pérez-López FR. Nutrition and health during mid-life: searching for solutions and meeting challenges for the aging population. Climacteric (2013) 16:85–95. 10.3109/13697137.2013.802884 23651240

[5] Bates Bea National Diet and Nutrition Survey Results From Years 5 and 6 (Combined) of the Rolling Programme (2012/2013 −2013/2014). London: TSO (2016).

[6] Yang M Chun OK. Consumptions of plain water, moisture in foods and beverages, and total water in relation to dietary micronutrient intakes and serum nutrient profiles among US adults. Public Health Nutr. (2015) 18:1180–6. 10.1017/S136898001400007X 24507693 PMC10271495

[7] King DE Xiang J Brown A. Intake of key chronic disease-related nutrients among baby boomers. South Med J. (2014) 107:342–7. 10.14423/01.SMJ.0000450706.44388.45 24945165 PMC4122273

[8] Dictionary O Middle Age. Available online at: https://en.oxforddictionaries.com/definition/us/middle_age (2018).

[9] Derbyshire EJ Nutrition in the Childbearing Years. Chichester: Wiley-Blackwell (2011).

[10] Salthouse TA. When does age-related cognitive decline begin? Neurobiol Aging. (2009) 30:507–14. 10.1016/j.neurobiolaging.2008.09.023 19231028 PMC2683339

[11] Bates B Cox L Nicholson S National Diet and Nutrition Survey Results from Years 5 and 6 (combined) of the Rolling Programme (2012/2013 – 2013/2014). Section 7: Methodological issues and response rates. London: FSA/PHE (2016).

[12] NDNS Appendix A: Dietary Data Collection and Editing. London: PHE (2016).

[13] COMA Dietary Reference Values for Food Energy and Nutrients for the United Kingdom. London: HMSO (1991).

[14] PHE Government Dietary Recommendations: Government Recommendations for Energy and Nutrients for Males and Females Aged 1-18 Years and 19+ Years. London: PHE (2016).

[15] Nielsen FH. Magnesium deficiency and increased inflammation: current perspectives. J Inflamm Res. (2018) 11:25–34. 10.2147/JIR.S136742 29403302 PMC5783146

[16] Maguire D Neytchev O Talwar D McMillan D Shiels PG. Telomere homeostasis: interplay with magnesium. Int J Mol Sci. (2018) 19:E157. 10.3390/ijms19010157 29303978 PMC5796106

[17] Sommer A Vyas KS. A global clinical view on vitamin A and carotenoids. Am J Clin Nutr. (2012) 96:1204S−6S. 10.3945/ajcn.112.034868 23053551

[18] Conklin AI Forouhi NG Suhrcke M Surtees P Wareham NJ Monsivais P. Variety more than quantity of fruit and vegetable intake varies by socioeconomic status and financial hardship. Findings from older adults in the EPIC cohort. Appetite (2014) 83:248–55. 10.1016/j.appet.2014.08.038 25195083 PMC4217146

[19] Colagar AH Marzony ET Chaichi MJ. Zinc levels in seminal plasma are associated with sperm quality in fertile and infertile men. Nutr Res. (2009) 29:82–8. 10.1016/j.nutres.2008.11.007 19285597

[20] Chasapis CT Loutsidou AC Spiliopoulou CA Stefanidou ME. Zinc and human health: an update. Arch Toxicol. (2012) 86:521–34. 10.1007/s00204-011-0775-1 22071549

[21] Olza J Aranceta-Bartrina J Gonzalez-Gross M Ortega RM Serra-Majem L Varela-Moreiras G. Reported dietary intake and food sources of zinc, selenium, and vitamins a, e and c in the spanish population: findings from the ANIBES Study. Nutrients (2017) 9:E697. 10.3390/nu9070697 28684689 PMC5537812

[22] Szabo Z Erdelyi A Gubicskone Kisbenedek A Ungar T Laszlone Polyak E Szekeresne Szabo S. [Plant-based diets: a review]. Orv Hetil. (2016) 157:1859–65. 10.1556/650.2016.30594 27868444

[23] Harris JK Duncan A Men V Shevick N Krauss MJ Cavazos-Rehg PA. Messengers and messages for tweets that used #thinspo and #fitspo hashtags in 2016. Prev Chronic Dis. (2018) 15:E01. 10.5888/pcd15.170309 29300696 PMC5757384

[24] Fayet-Moore F Petocz P Samman S. Micronutrient status in female university students: iron, zinc, copper, selenium, vitamin B12 and folate. Nutrients (2014) 6:5103–16. 10.3390/nu6115103 25401503 PMC4245582

[25] Bath SC Sleeth ML McKenna M Walter A Taylor A Rayman MP. Iodine intake and status of UK women of childbearing age recruited at the University of Surrey in the winter. Br J Nutr. (2014) 112:1715–23. 10.1017/S0007114514002797 25274294 PMC4340577

[26] Schupbach R Wegmuller R Berguerand C Bui M Herter-Aeberli I. Micronutrient status and intake in omnivores, vegetarians and vegans in Switzerland. Eur J Nutr. (2017) 56:283–93. 10.1007/s00394-015-1079-7 26502280

[27] Kristensen NB Madsen ML Hansen TH Allin KH Hoppe C Fagt S. Intake of macro- and micronutrients in Danish vegans. Nutr J. (2015) 14:115. 10.1186/s12937-015-0103-3 26518233 PMC4628270

[28] Derbyshire E. Associations between red meat intakes and the micronutrient intake and status of UK females: a secondary analysis of the UK National diet and nutrition survey. Nutrients (2017) 9:E768. 10.3390/nu9070768 28718824 PMC5537882

[29] Barker M Lawrence W Crozier S Robinson S Baird J Margetts B. Educational attainment, perceived control and the quality of women's diets. Appetite (2009) 52:631–6. 10.1016/j.appet.2009.02.011 19501760

[30] Leech RM Livingstone KM Worsley A Timperio A McNaughton SA. Meal frequency but not snack frequency is associated with micronutrient intakes and overall diet quality in australian men and women. J Nutr. (2016) 146:2027–34. 10.3945/jn.116.234070 27581583

[31] SACN The Nutritional Wellbeing of the British Population. London: TSO (2008).

[32] Speckmann B Grune T. Epigenetic effects of selenium and their implications for health. Epigenetics (2015) 10:179–90. 10.1080/15592294.2015.1013792 25647085 PMC4623467

[33] Roman M Jitaru P Barbante C. Selenium biochemistry and its role for human health. Metallomics (2014) 6:25–54. 10.1039/C3MT00185G 24185753

[34] Rayman MP Bath SC Westaway J Williams P Mao J Vanderlelie JJ. Selenium status in U.K. pregnant women and its relationship with hypertensive conditions of pregnancy. Br J Nutr. (2015) 113:249–58. 10.1017/S000711451400364X 25571960 PMC4302388

[35] Dama M Van Lieshout RJ Mattina G Steiner M. Iron deficiency and risk of maternal depression in pregnancy: an observational study. J Obstet Gynaecol Can. (2018) 40:698–703. 10.1016/j.jogc.2017.09.027 29307706

[36] Weaver CM. Potassium and health. Adv Nutr. (2013) 4:368S−77S. 10.3945/an.112.003533 23674806 PMC3650509

[37] White DJ Cox KH Peters R Pipingas A Scholey AB. Effects of four-week supplementation with a multi-vitamin/mineral preparation on mood and blood biomarkers in young adults: a randomised, double-blind, placebo-controlled trial. Nutrients (2015) 7:9005–17. 10.3390/nu7115451 26529011 PMC4663579

[38] Wallace TC McBurney M Fulgoni VL 3rd. Multivitamin/mineral supplement contribution to micronutrient intakes in the United States, 2007-2010. J Am Coll Nutr. (2014) 33:94–102. 10.1080/07315724.2013.846806 24724766

[39] Mistry HD Broughton Pipkin F Redman CW Poston L. Selenium in reproductive health. Am J Obstet Gynecol. (2012) 206:21–30. 10.1016/j.ajog.2011.07.034 21963101

[40] Mensink GB Fletcher R Gurinovic M Huybrechts I Lafay L Serra-Majem L. Mapping low intake of micronutrients across Europe. Br J Nutr. (2013) 110:755–73. 10.1017/S000711451200565X 23312136 PMC3785176

[41] Lentjes MA McTaggart A Mulligan AA Powell NA Parry-Smith D Luben RN. Dietary intake measurement using 7 d diet diaries in British men and women in the European Prospective Investigation into Cancer-Norfolk study: a focus on methodological issues. Br J Nutr. (2014) 111:516–26. 10.1017/S0007114513002754 24041116

[42] Miller R Spiro A Stanner S Micronutrient status and intake in the UK – where might we be in 10 years' time? Nutrition Bulletin. (2016) 41:14–41. 10.1111/nbu.12187

[43] WHO Micronutrients. Generva. Available online at: http://www.who.int/nutrition/topics/micronutrients/en/ (2018).

[44] PHE Public Health England Publishes New Advice on Vitamin D. London Available online at: https://www.gov.uk/government/news/phe-publishes-new-advice-on-vitamin-d (2016).

[45] Hibberd MC Wu M Rodionov DA Li X Cheng J Griffin NW. The effects of micronutrient deficiencies on bacterial species from the human gut microbiota. Sci Transl Med. (2017) 9:eaal4069. 10.1126/scitranslmed.aal4069 28515336 PMC5524138

[46] Ji X Grandner MA Liu J. The relationship between micronutrient status and sleep patterns: a systematic review. Public Health Nutr. (2017) 20:687–701. 10.1017/S1368980016002603 27702409 PMC5675071

